# Transitional Probability-Based Model for HPV Clearance in HIV-1-Positive Adolescent Females

**DOI:** 10.1371/journal.pone.0030736

**Published:** 2012-01-24

**Authors:** Julia Kravchenko, Igor Akushevich, Staci L. Sudenga, Craig M. Wilson, Emily B. Levitan, Sadeep Shrestha

**Affiliations:** 1 Duke Cancer Institute, Duke University Medical Center, Duke University, Durham, North Carolina, United States of America; 2 Center for Population Health and Aging, Duke University, Durham, North Carolina, United States of America; 3 Department of Epidemiology, University of Alabama at Birmingham, Birmingham, Alabama, United States of America; Instituto de Pesquisa Clínica Evandro Chagas/Fundação Oswaldo Cruz, Brazil

## Abstract

**Background:**

HIV-1-positive patients clear the human papillomavirus (HPV) infection less frequently than HIV-1-negative. Datasets for estimating HPV clearance probability often have irregular measurements of HPV status and risk factors. A new transitional probability-based model for estimation of probability of HPV clearance was developed to fully incorporate information on HIV-1-related clinical data, such as CD4 counts, HIV-1 viral load (VL), highly active antiretroviral therapy (HAART), and risk factors (measured quarterly), and HPV infection status (measured at 6-month intervals).

**Methodology and Findings:**

Data from 266 HIV-1-positive and 134 at-risk HIV-1-negative adolescent females from the Reaching for Excellence in Adolescent Care and Health (REACH) cohort were used in this study. First, the associations were evaluated using the Cox proportional hazard model, and the variables that demonstrated significant effects on HPV clearance were included in transitional probability models. The new model established the efficacy of CD4 cell counts as a main clearance predictor for all type-specific HPV phylogenetic groups. The 3-month probability of HPV clearance in HIV-1-infected patients significantly increased with increasing CD4 counts for HPV16/16-like (p<0.001), HPV18/18-like (p<0.001), HPV56/56-like (p = 0.05), and low-risk HPV (p<0.001) phylogenetic groups, with the lowest probability found for HPV16/16-like infections (21.60±1.81% at CD4 level 200 cells/mm^3^, p<0.05; and 28.03±1.47% at CD4 level 500 cells/mm^3^). HIV-1 VL was a significant predictor for clearance of low-risk HPV infections (p<0.05). HAART (with protease inhibitor) was significant predictor of probability of HPV16 clearance (p<0.05). HPV16/16-like and HPV18/18-like groups showed heterogeneity (p<0.05) in terms of how CD4 counts, HIV VL, and HAART affected probability of clearance of each HPV infection.

**Conclusions:**

This new model predicts the 3-month probability of HPV infection clearance based on CD4 cell counts and other HIV-1-related clinical measurements.

## Introduction

HIV-1-positive women clear HPV infections 4–10 times more slowly than HIV-1-negative, and HIV-1-infected patients with CD4+ T-lymphocytes cell count (CD4) <200 cells/mm^3^ show the slowest clearance [1], [2]. Understanding the role of immunosuppression in risk of persistence of sexually transmitted human papillomavirus (HPV) infection, a main risk factor for cervical intraepithelial neoplasia and a central etiologic agent of cervical cancer, and clarifying how this risk is modified by other factors (such as co-infections, antiretroviral therapy, and behavioral factors) is important for optimization of follow-up strategy [3]. Information from longitudinal studies about factors that affect the probability of type-specific HPV clearance can be used to estimate the impact of cervical cancer interventions. However, this is often problematic because HPV persistence is loosely defined as detection of the same HPV type at two or more subsequent visits, ranging from 2 months to 7 years [4], [5], [6], [7], [8], [9], and its probability depends on intervals between the tests. Further, the analysis is complicated by the possibility of co-infections with multiple HPV types as well as by varying length of intervals between missing measurements of HPV status. Analytical methods that can fully utilize real-life heterogeneous data, specifically, clinical data that are unevenly scheduled (for example, at 6 months for HPV and at 3-month intervals for HIV clinical data) could be useful in studies of the factors having an impact on probability of clearance of HPV infection.

Here, we describe a transition probability model for studying the relationship between immune status (based on CD4 cell count) and probability of HPV clearance in HIV-infected patients. The Reaching for Excellence in Adolescent Care and Health (REACH) dataset used in this study planned for measurement of HIV-1 status every 3 months and HPV status every 6 months. As in other cohorts, this study had missing and irregular visit measurements: only 82% of time intervals between measurements of HPV statuses were performed as scheduled (i.e., every 6 months), while other HPV tests were done at 3-, 9-, 12-, or other-month intervals. Analyzing these data with standard techniques would require multiple assumptions about the definition of HPV clearance and censoring time for each individual for time-dependent predictors; as a result, part of the dataset would not be utilized. We have developed transition probability-based models and applied an HPV/HIV co-infected cohort to estimate 3-month HPV clearance probabilities while maximizing all available data for both HIV and HPV in the estimations.

## Materials and Methods

Data on 266 HIV-1-positive and 134 high-risk HIV-1-negative adolescent females from the REACH cohort were analyzed. The REACH study design and methods of quarterly follow up with HIV-1 testing, immunophenotyping, HIV-1 RNA viral load (VL) and collection of biological specimens, demographic and behavioral factors, and other clinical data, along with incidence and prevalence of HPV infections, have been previously described [2], [10], [11]. Briefly, between 1996 and 2000, adolescents aged 12–19 years who were HIV-1-positive and comparable at-risk HIV-1-negative persons were recruited into a longitudinal study at 15 clinical sites in the United States. HIV-1-related clinical data and risk factors were measured every 3 months. At enrollment and every 6 months thereafter, cervical lavage samples were tested for HPV infection by MY09/MY11/HMB01-based PCR and for 30 HPV type-specific probes with a chemiluminescent dot blot procedure [12]. PCR-based HPV data were classified as follows: negative; positive for specific HPV types; or “positive, type unknown” (when the sample was positive for the generic probe but not for specific HPV type). PCR amplification of a human β-globin gene segment was used for internal DNA quality control, and samples negative for this assay were excluded from the analysis. For certain types of analyses, HPVs were categorized according to phylogenetic patterns [2], [13] into: 1) 16/16-like (16, 31, 52, 58, 67); 2) 18/18-like (18, 39, 45, 59, 26, 51); 3) 56/56-like (56, 53, 66); and 4) low-risk (6, 11, 42, 44, 54, 40, 13, 32, 62, 72, 2, 57, 55). The highly active antiretroviral therapy (HAART) was defined as a combination of two nucleoside reverse transcriptase inhibitors and either a protease inhibitor (PI) or a non-nucleoside reverse transcriptase inhibitor, or a zidovudine/lamivudine combination regimen plus another antiretroviral drug. Data on antiretroviral therapy were obtained through interviews and chart reviews for current prescriptions, and adherence data were obtained through interviews as previously described [14].

### Ethics Statement

All the participants of the study provided written informed consent in the parent study at the Adolescent Medicine HIV/AIDS Research Network for the REACH project, and the UAB Institutional Review Board approved this sub-study. The parent study and this sub-study conformed to the procedures for informed consent (parental permission was obtained wherever required) approved by institutional review boards at all sponsoring organizations and to human-experimentation guidelines set forth by the United States Department of Health and Human Services.

*Cox proportional hazard* regression was used to test which covariates have significant effects on probability of HPV clearance for each of four phylogenetic HPV groups. First, an univariable regression analysis was used for a wide spectrum of variables including demographic characteristics, clinical exams, antiretroviral therapy, behavioral factors, and coinfections (as listed in Table 1). From this, a subset of predictors with significant effects was selected and considered in multivariable analysis. For this analysis, SAS PROC PHREG (Cary, NC) was used.

**Table 1:** Demographic, behavioral, and clinical characteristics of adolescent female study participants from the REACH cohort.

Variable	HIV-1-positive	HIV-1-negative	OR (95% CI)
**Number of patients**	N = 262	N = 134	-
**Age, years^1^**	16.8 (1.1)	16.6 (1.2)	-
**Number of visits per patient^1^**	11.0 (4.88)	8.7 (4.36)†	-
**Number of visits per patient with measured HPV status^1^**	5.6 (2.5)	4.7 (2.6) †	-
**Race^2^**African AmericansCaucasiansOthers	206 (78.6%)15 (5.7%)41 (15.6)%	92 (68.7%)12 (9.0%)30 (22.4%)	1.79 (0.81–3.98)‡Referent1.09 (0.45–2.67)
**Baseline CD4+ T cell count, cells/mm^3 1^**	535.2 (263.6)	896.5 (258.9)†	-
**Number of lifetime sexual partners^2^**<66–15>15	81 (30.9%)104 (39.7%)77 (29.4%)	54 (40.3%)43 (32.1%)37 (27.6%)	Referent1.61 (0.98–2.85)1.39 (0.82–2.34)
**Ever smoked cigarettes^2^**Never smokedSmoked (≥100 cigarettes)	33 (12.6%)205 (78.2%)	20 (14.9%)101 (75.4%)	0.81 (0.44–1.49)Referent
**Trichomonas infection^2^**NegativePositive	219 (83.6%)34 (13.0%)	128 (95.5%)†2 (1.5%)	Referent9.93 (2.35–42.03) ‡
**Gonorrhea infection^2^**NegativePositive	208 (79.4%)22 (8.4%)	104 (77.6%)9 (6.7%)	Referent1.22 (0.54–2.75)
**Chlamydia infection^2^**NegativePositive	182 (69.5%)50 (19.1%)	90 (67.2%)26 (19.4%)	Referent0.95 (0.56–1.62)
**HIV VL, logarithm ^1^**	3.44 (1.01)	-	-
**Currently taking ART medications^2^**Not on ART drugsMono or combination therapy without PICombo therapy with PI	125 (47.7%)101 (38.5%)35 (13.4%)	-	-
**ART therapy ever used^2^**No ART was usedMono or combination therapy without PICombo therapy with PIART regimen unknown	98 (37.4%)105 (40.0%)57 (21.8%)2 (0.8%)	-	-

Notes: ^1^ – results are presented as mean (SD); ^2^ – number of cases (percent);

† – p<0.05 for the difference between HIV-1-positive and HIV-1-negative: continuous variables were analyzed by general linear model, and categorical were analyzed by chi-square;

‡ – p<0.05 for the difference with the referent group; continuous variables were analyzed by general linear model, and categorical were analyzed by PROC LOGISTIC.

The model developed in the present paper falls under the general category of transitional Markov models and is referred to as a *transitional probability-based model*. These kinds of approaches, also known as regressive or conditional models, are used in epidemiology for analyses of dependent binary observations [15], [16], [17]. The model allows for estimating the probability of changing HPV status in patients with specific HPV type, i.e., a chance to clear HPV infection during the follow-up period. To account for HIV-1-related clinical data such as CD4 counts, HIV-1 VL, and other HIV-1-related factors, which were measured every 3 months, HPV infection status was reconstructed for the same time interval considering transition probabilities. The transition probabilities were referred to as 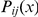, where 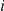 was initial state of type-specific HPV infection (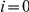 corresponded to absence, and 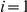
to presence of type-specific HPV infection), and 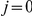 and 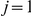 corresponded to the status of type-specific HPV infection at the end of a 3-month period. Vector 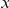 denoted the set of most influential predictors of HPV clearance probability, such as CD4 count, HIV-1 VL, HAART, and HPV type [2], [18]. For example, the probability 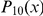 corresponded to the situations where HPV infection observed at a recent visit was cleared in 3 months.

**Figure 1:**
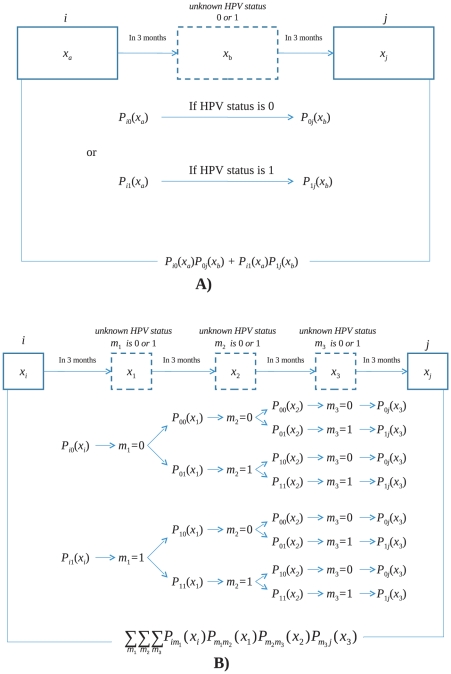
Reconstruction of information about the missed measurements when one HPV status is unknown (**Figure 1A**) or several (e.g., three) HPV statuses in a raw are missed (**Figure 1B**). Here, 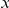 denotes the set of predictors of HPV clearance probability, such as CD4 count, HIV-1 VL, HAART, and HPV type. When one HPV measurement is unknown (Figure 1A), *i* and *j* describe the HPV status at the first and third visits, respectively, and parameters 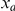 and 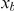 denote the sets of predictors for transitions between first-to-second and second-to-third visits, respectively. The probability of changing HPV status from the first (i.e., known) state of HPV infection *i* to the status of HPV infection at the second visit (i.e., unknown) is *P_i_*_0_(*x_a_*) when HPV status at the second visit is negative (i.e., “0”) or *P_i_*_1_(*x_a_*) when it is positive (i.e., “1”). Respectively, at the third visit (with measured/known HPV status) HPV status *j* can be defined as *P*_0*j*_(*x_b_*) when at the second visit it supposed to be HPV-negative, and *P*_1*j*_(*x_b_*) when at the second visit it supposed to be HPV-positive. The sum over two possible intermediate states contributes to the total transition probability: so, the transition probability between two subsequent visits with measured HPV status could be presented as 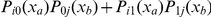. When three subsequent HPV status are unknown (Figure 1B), there are eight different combinations of HPV statuses in these states, each denoted by 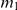, 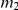, and 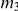 as unmeasured HPV statuses which can be 0 or 1). Therefore, the transition probability between states with known HPV statuses is calculated as three-fold sum over all combinations of HPV statuses in these three unmeasured states.

With this model, the information about missed measurements at odd visits can be reconstructed using the previous and forthcoming measurements (see Figure 1A). The transition probability between two subsequent visits with measured HPV status could be presented as 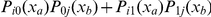, where *i* and *j* describe the HPV status at the first and third visits, respectively. The status at a second visit is unknown, and, therefore, the sum over two possible intermediate states contributes to the observed transition probability between two subsequent even visits. Parameters 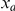 and 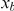 in the above formula denote the sets of respective predictors for transitions between first-to-second and second-to-third visits, respectively. The likelihood function is the product over all transfers with known HPV status. If data are taken exactly according to the cohort's measurement design, the likelihood is
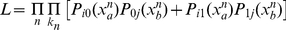
(1)Here, 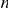
covers all individuals in the dataset, 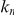
—all transitions between states with measured HPV virus status represented by indices 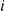 and 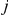 in individual
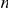
_,_ and 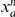 and 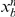 are the vectors of predictors measured at the beginning of time period of respective transition. The dependence of transition probabilities on predictors are modeled in logistic regression style as:
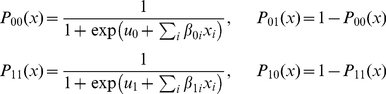
(2)where intercepts 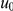 and 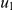 refer to logarithms of odds of changing the type-specific HPV status for zeroth values of predictors, and parameters 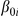 and 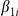 describe the effects of respective predictors [19].

If the number of missed HPV status is 0 (zeroth) or varies from two to five, the likelihood function could be generalized by summing over all intermediate states. For example, for transitions with three missed HPV statuses (see Figure 1B), the contribution to the likelihood function could be presented as:
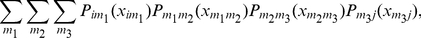
(3)where 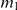, 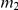, and 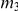 are unmeasured (0 or 1) HPV status in three intermediate states. The set of observed transfers with measured HPV statuses for a specific type of HPV virus or for HPV group is the input dataset for likelihood maximization. Note that CD4 count and HIV-1 VL also could have missing measurements; however, the appearance of these missing variables is not related to the study design, so we assume that they are missing at random, and any standard approach for filling missing data at random can be applied (e.g., imputation with the mean conditional on observed values of other variables or simply linear interpolation). Because in the REACH cohort, the fractions of missing values of predictors of HPV clearance were relatively small (about 2% for CD4 count in HIV-1-positive patients, about 5% for HIV-1 VL, and about 1% for HAART), no notable impact of specific scheme of their filling was expected. The reported results were obtained using a linear interpolation to fill missing values in the predictors (but not HPV) status.

To test the different hypotheses regarding the possible effects of different potential predictors on probability of HPV clearance, a two-stage approach was designed. The two-stage approach complemented the advantages of methods used at each stage such as nonparametric estimates of hazard ratios in the Cox model and opportunities for evaluating 3-month probabilities for models (1) – (3). The comparison of the results obtained from both approaches allows for validating the properties of the new method. At the first stage, the effects were evaluated using the Cox proportional hazard model, and then the variables that demonstrated significant effects on HPV clearance in the Cox analysis were included in the logistic type models (substantively specified below). Note, the results can be obtained only under specific assumptions that are necessary to identify the times of HPV incidence and clearance/censoring. Moreover, the specific assumptions also are required to decide how to treat missing data on unknown HPV status. There is no consensus in literature about the choice of specific assumptions in these situations. Based on the published studies, our set of assumptions included: 1) time of HPV incidence was 90 days before the first exam with positive HPV status; 2) patient was removed when a time period between measurements exceeded 450 days; 3) when no time period between measurements exceeded 450 days, the clearance time was the time of the first (of the two subsequent) negative exams, ignoring any missing visits; 4) when the last exam was positive, the censoring time was the time of this exam plus 180 days; and 5) when the last exam was negative (after the positive), the censoring time was the time of this exam.

The approach used at the second stage (i.e., the formalism based on eq. (1–3)) does not require these assumptions. All logistic-type models described the probability of a 3-month HPV clearance for different predictors using the base equation 2; their parameters were estimated using the likelihood described in equation 1 and generalized in the style of equation 3. The CD4 count parameter was selected to be tested by the model as a main predictor of HPV clearance in HIV-1-positive patients based on multiple studies [2], [20], [21]—the base model (M1) had it as a main parameter and an argument of the exponent for the model in equation 2 was 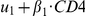. Then, additional models were developed, extending the base model as follows:

M2 —includes the effect of the presence of HIV-1 infection on intercept of 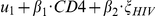, where 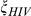 is the binary variable characterizing the HIV-1 seropositivity (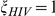) or HIV-1 seronegativity (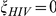). While the base model M1 is applied separately for HIV-1-positive and HIV-1-negative patients, the M2 as well as the M3 models are designed to investigate whether and how specifically the effect of CD4 counts on HPV clearance probability is influenced by the presence of HIV-1 infection, which may have an effect on HPV clearance beyond the CD4 counts effect (e.g., this effect could be further compared with the patients with other immunodeficiencies such as those with organ transplants or inherited immune disorders).

M3 —extends the M2 model by including the possible interference of the effects of HIV-1 seropositivity and CD4 count on HPV clearance 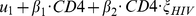. This model evaluates the modifying effect of CD4 on HPV clearance by the presence of HIV-1-infection.

M4 —in HIV-1-infected patients, it introduces CD4 count as a squared parameter in 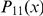 (the exponent argument is 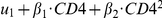) to test the assumption about non-linearity (squared CD4) in interrelations between CD4 count and HPV clearance probability in HIV-1-positive patients.

M5 —in HIV-1-positive patients, the effect of CD4 count is described as a piecewise-linear interpolation with an arbitrary set 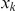, 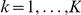 of 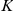 nodes (i.e., CD4 count values at which the linear functions are joined):
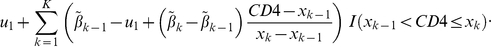



where 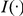 is the indicator function and 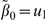; parameters 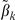 referring to the logarithms of odds of changing the HPV status for 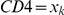. This model tests whether different shapes of interrelations between CD4 counts and probability of HPV clearance are possible in HIV-1-positive patients at a very low (<200 cells/mm^3^), low (200–499 cells/mm^3^), and normal range of CD4 counts.

M6 —includes the logarithm of HIV-1 VL (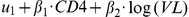) to evaluate the effect of HIV-1 VL on probability of HPV clearance in HIV-1-positive patients controlling the level of CD4 count.

M7 —investigates the effects of HAART that include PIs on the intercept 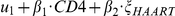, where 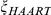 is a binary variable of HAART; 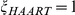 when HAART (with PI) was applied at the time of the visit. This model tests whether HAART with PIs may have an additional effect on interrelations between CD4 count and probability of HPV clearance in HIV-1-infected patients.

The nonlinear optimization techniques as implemented in PROC NLP in SAS 9.2 (Cary, NC) were used for the likelihood maximization in all these models to estimate a 3-month probability of HPV clearance. For the majority of calculations, the intercept and the effects of the predictors were chosen as model parameters and estimated with the standard errors (SEs). Because of the functional relation between parameter *β* (which describes the effects of predictor of clearance) and the clearance probability for a certain value of predictor, the latter can be used as a model parameter instead of *β*. Estimation of this model using the Proc NLP allows for evaluating its standard error.

## Results

The main characteristics of studied patients are listed in Table 1. The average age of HIV-1-infected adolescent females were 16.8 (±1.1) years old, and 78.6% of them were African Americans. On average, they had about 11 HIV-1 status-related visits/examinations per patient (quarterly); however, HPV data were collected only biannually, so during half of these visits. Table 2 provides detailed descriptions of incident and prevalent HPV infections: among oncogenic HPVs, HPV16, 31, 52, and 67 (HPV16/16-like group), HPV59 and 26 (HPV18/18-like group), and HPV56 and 53 (HPV56/56-like group) were more often (p<0.05) registered in HIV-1-infected than HIV-1-negative patients.

**Table 2:** Incident and prevalent HPV infection, by subtype, in the REACH cohort.

Variable		HIV-1-positive (n = 262)			HIV-1-negative (n = 134)		
HPV infection		Non-infected	Prevalent infection	Incident infection	Non-infected	Prevalent infection	Incident infection
**HPV16/16-like**	HPV16	177 (67.6%)	45 (17.2%)	40 (15.3%)	108 (80.6%)	7 (5.2%)	19 (14.2%)
	HPV31/33/35	166 (63.4%)	39 (14.9%)	57 (21.8%)	105 (78.4%)	12 (9.0%)	17 (12.7%)
	HPV52	197 (75.2%)	31 (11.8%)	34 (13%)	117 (87.3%)	4 (3.0%)	13 (9.7%)
	HPV58	182 (69.5%)	43 (16.4%)	37 (14.1%)	102 (76.1%)	12 (9.0%)	20 (14.9%)
	HPV67	240 (91.6%)	2 (0.8%)	20 (7.6%)	131 (97.8%)	1 (0.7%)	2 (1.5%)
**HPV18/18-like**	HPV18	199 (76.0%)	20 (7.6%)	43 (16.4%)	112 (83.6%)	10 (7.5%)	12 (9.0%)
	HPV39	232 (88.5%)	11 (4.2%)	19 (7.3%)	128 (95.5%)	1 (0.7%)	5 (3.7%)
	HPV45	218 (83.2%)	13 (5.0%)	31 (11.8%)	119 (88.8%)	3 (2.2%)	12 (9.0%)
	HPV51	221 (84.4%)	7 (2.7%)	34 (13.0%)	115 (85.8%)	4 (3.0%)	15 (11.2%)
	HPV59/68/70	170 (64.9%)	28 (10.7%)	64 (24.4%)	105 (78.4%)	7 (5.2%)	22 (16.4%)
	HPV26/69	221 (84.4%)	6 (2.3%)	35 (13.4%)	124 (92.5%)	3 (2.2%)	7 (5.2%)
**HPV56//56-like**	HPV56	215 (82.1%)	20 (7.6%)	27 (10.3%)	123 (91.8%)	3 (2.2%)	8 (6.0%)
	HPV53/66	142 (54.6%)	40 (15.3%)	79 (30.2%)	103 (76.9%)	5 (3.7%)	26 (19.4%)
**HPV low-risk**	HPV6/11/42/44	178 (67.9%)	32 (12.2%)	52 (19.8%)	109 (81.3%)	11 (8.2%)	14 (10.4%)
	HPV54/40	191 (72.9%)	10 (3.8%)	61 (23.3%)	114 (85.1%)	4 (3.0%)	16 (11.9%)
	HPV13/32	222 (84.7%)	2 (0.8%)	38 (14.5%)	127 (94.8%)	1 (0.7%)	6 (4.5%)
	HPV62/72	224 (85.5%)	5 (1.9%)	33 (12.6%)	128 (95.5%)	1 (0.7%)	5 (3.7%)
	HPV2/57	252 (96.2%)	1 (0.4%)	9 (3.4%)	134 (100%)	0	0
	HPV55	250 (95.4%)	0	12 (4.6%)	131 (97.8%)	0	3 (2.2%)

Notes: results are presented as number of cases (percent);

† – p<0.05 for the difference between HIV-1-positive and HIV-1-negative; categorical variables were analyzed by chi-square.

The results of the Cox proportional hazard analysis for HIV-1-infected adolescent females are shown in Table 3. No significant HRs were obtained for such parameters as having <6 or >15 lifetime sexual partners, ever being a smoker, or being infected with *Trichomonas vaginalis* or *Neisseria gonorrhea*. CD4 count was a consistent predictor for clearance of HPV16/16-like, 18/18-like, and low-risk groups. For these HPV groups, a significantly higher probability of HPV clearance was at CD4 levels higher than 500 cells/mm^3^ (compared with those at <500 cells/mm^3^); the difference was especially pronounced for CD4 categorical cutout at 200 cells/mm^3^. For HIV-1 VL, significant effect on HPV clearance (for all except HPV56/56-like group) was observed only without CD4 count as a second parameter (in the univariable analysis). While being on HAART was a significant predictor of HPV16/16-like clearance only, being on HAART with PI significantly increased probability of clearance of 16/16-like, 18/18-like, and low-risk HPV infection. Also, being infected with *Chlamydia trachomatis* could be a positive predictor for low-risk HPV clearance.

**Table 3:** Hazard ratios for HPV infection clearance probability for HIV-1-infected adolescent females from the REACH cohort, univariable and multivariable Cox proportional hazard regression (results are presented with 95% CIs).

Parameter	HPV16/16-like		HPV18/18-like		HPV56/56-like		HPV low risk	
	Univariable	Multivariable	Univariable	Multivariable	Univariable	Multivariable	Univariable	Multivariable
**CD4/100 (per each 100cells/mm^3^ increase)**	1.08(1.06,1.10)^‡^	1.15(1.08,1.23)^†^	1.05(1.03,1.08)^†^	1.34(1.24,1.45)^‡^	ns	ns	1.11(1.08,1.14)^‡^	1.24(1.12,1.36)^†^
**CD4 level** ≥**200 cells/mm^3^ (vs. <200 cells/mm^3^)**	1.68(1.32,2.14)^†^	n/a	1.80(1.40,2.31)^†^	n/a	ns	n/a	2.53(1.81,3.53)^†^	n/a
**CD4 level** ≥**500 cells/mm^3^ (vs. <500 cells/mm^3^)**	1.65(1.42,1.91)^‡^	n/a	1.70(1.45,1.98)^‡^	n/a	ns	n/a	2.11(1.74,2.57)^‡^	n/a
**HIV-1 VL**	0.82(0.76,0.88)^†^	ns	0.79(0.73,0.86)^‡^	ns	ns	ns	0.74(0.67,0.81)^†^	ns
**HAART**	ns	1.42(1.22,1.66)^†^	ns	ns	ns	ns	ns	ns
**HAART with PI**	1.57(1.33,1.84)^†^	1.77(1.50,2.08)^‡^	1.75(1.47,2.08)^‡^	1.79(1.50,2.13)^‡^	ns	ns	1.68(1.36,2.08)^†^	1.82(1.47,2.27)^†^
**Any HPV infection at baseline**	ns	ns	ns	ns	ns	ns	ns	ns
***Chlamydia trachomatis***	ns	ns	ns	ns	ns	ns	1.69(1.33,2.14)^†^	1.60(1.26,2.02)^†^

Note: ^†^p<0.05; ^‡^p<0.001; ns - not significant; n/a – not applicable.

Sensitivity analysis was performed to check the stability of HRs estimated by the Cox model. In many cases, the HRs essentially shifted when assumptions changed: e.g., the effect of CD4 being >200 cells/mm^3^ changed for HPV16/16-like infection from 1.68 to 1.86, and from 2.53 to 3.07 (still remaining significant) when 365 days were used in assumptions #2 and #3 instead of 450 days. Another example is the changes of the HAART effect from 1.77 to 2.03 for HPV16/16-like infection, which remained highly significant while using 270 days instead of 450 in these assumptions.

**Figure 2:**
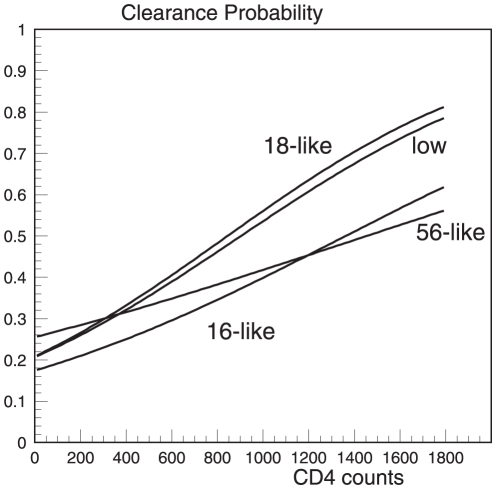
The 3-month HPV type-specific probability of clearance depending on CD4 T-lymphocytes in HIV-1-positive adolescent girls from the REACH cohort.

The results obtained from the basic model and from the models describing the effects of HIV-1 VL and HAART (with PIs) are shown in Table 4, and the results obtained from all models (M1–M7) are presented in Table S1. The probability of HPV clearance in HIV-1-infected patients increased with increasing CD4 level for all HPV groups: parameter *β_11_* (the log odds ratio, describing the effect of CD4 count on HPV clearance) was 1.15 (0.27) for HPV16/16-like (p<0.001), 1.58 (0.36) for HPV18/18-like (p<0.001), 0.72 (0.38) for HPV56/56-like (p = 0.059), and 1.5 (0.41) for low-risk HPVs (p<0.001). HPV16/16-like infection was least likely to clear at low CD4 cell count (<200 cells/mm^3^) than other HPV groups (see Figure. 2): a probability of HPV16/16-like clearance was 21.60 (1.81)% vs. 27.40 (2.38)% for HPV18/18-like, 29.96 (3.30)% for HPV56/56-like, and 26.60 (2.79)% for low-risk HPVs (see Table 5). The interrelations between probability of clearance of oncogenic HPVs and CD4 likely had a piecewise shape for CD4 count <500 cells/mm^3^ (see Table S1). The effect of HIV-1 VL was minor on HPV16/16-like clearance (p = 0.061) and significant for low-risk HPV (p<0.05) (the M6 model, Table 4). A minor effect of HAART with PIs was observed on HPV16/16-like (p = 0.060) clearance (the M7 model, Table 4).

**Table 4:** CD4 T-lymphocyte counts (basic model M1), HIV VL (M6 model), and HAART with PI (M7 model) effects on probability of HPV clearance, by phylogenetic HPV group, in HIV-1-infected adolescent females, REACH cohort.

HPV type	Model	*u_00_* (SE)	*β_00_* (SE)^a^	*u_11_* (SE)	*β_11_* (SE)^a^	Additional parameter in the model (SE)
**16/16-like**	M1	−3.5±0.15**	−0.14±0.25	−1.52±0.15**	1.15±0.27**	––
	M6	−3.47±0.17**	−0.09±0.28	−0.7±0.47*	0.79±0.35**	−0.17±0.09*
	M7	−3.5±0.14**	−0.13±0.25	−1.65±0.17**	1.23±0.27**	0.33±0.18*
**18/18-like**	M1	−3.38±0.14**	−0.47±0.25*	−1.29±0.18**	1.58±0.36**	––
	M6	−3.48±0.16**	−0.2±0.28	−0.98±0.64*	2.1±0.66**	−0.14±0.12
	M7	−3.38±0.13**	−0.47±0.25**	−1.38±0.18**	1.58±0.36**	0.29±0.21*
**56/56-like**	M1	−2.82±0.19**	−0.59±0.35*	−0.99±0.22**	0.72±0.38*	––
	M6	−3.05±0.24**	−0.35±0.4	−0.90±0.67*	0.7±0.51	−0.05±0.13
	M7	−2.82±0.19**	−0.58±0.35*	−1.03±0.25**	0.74±0.38**	0.09±0.25
**Low risk**	M1	−3.69±0.15**	−0.26±0.27	−1.31±0.21**	1.5±0.41**	––
	M6	−3.82±0.17**	0.05±0.29	−0.13±0.64	0.93±0.51*	−0.26±0.13**
	M7	−3.69±0.15**	−0.27±0.27	−1.42±0.22**	1.54±0.41**	0.33±0.24

Note:

*0.05≤p<0.1;

**p<0.05.*u_00_*, *β_00,_ u_11,_ and β_11_* are related to the parameters in equation (2)

a – the units of *β_00_* and *β_11_* are 1000/[C], where [C] are the units of CD4 cell counts, i.e., cells/mm^3^.

SEs were obtained by re-estimating the model in which probability at specific value of CD4 cell count was chosen as a model parameter instead of 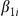.

**Table 5:** Probability of HPV clearance (in %, ±SE) at specific CD4 levels, by phylogenetic HPV group, in HIV-1-positive adolescent females, REACH cohort.

CD4 cell (cells/mm^3^)	HPV16/16-like	HPV18/18-like	HPV56/56-like	Low-risk HPV
**200**	21.60±1.81	27.40±2.38*	29.96±3.30*	26.60±2.79*
**500**	28.03±1.47	37.77±2.08*	34.66±2.51*	36.24±2.50*
**750**	34.19±2.24	47.42±3.57*	38.83±3.50	45.26±4.11*
**1000**	40.93±3.69	57.27±5.44*	43.17±5.45	54.60±6.28*
**1500**	55.22±6.84	74.74±7.46*	52.09±9.95	71.80±9.06*

Note: *The difference with HPV16/16-like type is significant (p<0.05).

When two oncogenic groups of HPV infections—HPV16/16-like and HPV18/18-like—were analyzed for each HPV type separately, both groups showed heterogeneity in terms of how probability of type-specific HPV clearance was affected by CD4 counts, HIV VL, and HAART with PI (see Table 6): while lower clearance probability was registered for HPV16/16-like than for the 18/18-like group (p<0.05). Interestingly, HPV16 and HPV18 alone had an equal chance to be cleared at all CD4 levels examined: i.e., 20.29(3.76)% and 18.16(4.04)% at CD4 level 200 cells/mm^3^, 26.63(3.19)% and 28.66(4.0)% at CD4 level 400 cells/mm^3^, and 34.12(3.49)% and 42.12(6.24)% at CD4 level 600 cells/mm^3^, for HPV16 and HPV18, respectively). The effect of HIV-1 VL was significant for clearance of HPV58 (16-like group) and HPV59 (18-like group), and the effect of HAART (with PIs) was significant for HPV16 clearance. In average, HPV67 had a higher probability to be cleared than the other 16/16-like HPV types: e.g., 64.11 (15.72)% vs. 25.76 (1.48)% at CD4 level 400 cells/mm^3^ (p<0.05). Likewise, HPV18 had the lowest probability to be cleared (18.16 (4.04)%), and HPV26 the highest (42.12 (8.39)%) than other 18/18-like HPV types at CD4 level 200 cells/mm^3^ compared to the group at average (27.40 (2.38)%), p<0.05.

**Table 6:** CD4 T-lymphocyte counts (basic model M1), HIV VL (M6 model), and HAART (M7 model) effects on HPV clearance probability, HPV type-specific, in HIV-1-positive adolescent females, REACH cohort.

HPV type		M1 (basic model): CD4 effect		M6 model:HIV VL effect,	M7 model: HAART(PI) effect
		u_11_ (SE)	β_11_ (SE)^a^		
**HPV16/16-like group**	HPV16	−1.72(0.33)**	1.78(0.56)**	ns	0.99(0.38)**
	HPV31	−1.55(0.32)**	0.97(0.55)*	−0.14(0.17)	0.01(0.34)
	HPV52	−1.42(0.31)**	1.28(0.56)**	−0.25(0.2)	0.38(0.38)
	HPV58	−1.54(0.28)**	0.82(0.48)*	−0.53(0.22)**	0.001(0.39)
	HPV67	ns^b^	ns	−8.46(8.3)	0.65(1.16)
	HPV16/16-like	−1.52(0.15)**	1.15(0.27)**	−0.17(0.09)*	0.33(0.18)*
**HPV18/18-like group**	HPV18	−2.1(0.42)**	2.97(0.89)**	−0.37(0.28), p = 0.188	0.56(0.47)
	HPV39	−0.78(0.58)	1.97(1.24)	ns	0.06(0.75)
	HPV45	−1.66(0.5)**	3.03(1.13)**	0.03(0.24)	−0.26(0.57)
	HPV51	−1.41(0.53)**	1.61(1.05)	0.62(0.39)*	−0.15(0.71)
	HPV59	−1.12(0.31)**	0.86(0.56)*	−0.43(0.2)**	0.62(0.36)*
	HPV26	−0.49(0.51)	0.87(1.10)	−0.08(0.4)	0.22(0.65)
	HPV18/18-like	−1.29(0.18)**	1.58(0.36)**	−0.14(0.12)	ns

Note: * 0.05≤p<0.1; ** p<0.05. *u_00_*, *β_00,_ u_11,_ and β_11_* are related to the parameters in equation (2).

a – the units of *β_00_* and *β_11_* are 1000/[C], where [C] are the units of CD4 cell counts, i.e., cells/mm^3^.; b – non-significant.

SEs were obtained by re-estimating the model in which probability at specific value of CD4 cell count was chosen as a model parameter instead of 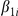.

## Discussion

The longitudinal studies of HPV infection are important for determining the covariates and outcomes associated with HPV persistence, which leads to the development of cancer. Traditionally, the rate of HPV clearance are usually compared in HIV-1-positive patient subgroups based on baseline CD4 counts (such as <200, 200–500, and >500 cells/mm^3^) using Kaplan-Meier curves and Cox proportional hazards models [1], [2], [22], [23]. The new method developed in this study is based on the logistic-type model and allows for prediction of future HPV status, conditional on its current status and the measurements of factors that are potential predictors of an HPV clearance event; i.e., CD4 count was considered to be a main predictor with HIV-1 VL and HAART (with PI) as additional predictors. The model parameters were estimated by maximizing the likelihood function constructed as the product over transfers with known HPV statuses (measured every 6 months) and HIV-1-related covariates (measured every 3 months). Similar to Kong et al. [17], our methodology is based on conditional probabilities that take into account multiple correlations between individual outcomes measured longitudinally. However, the developed method extends the opportunities of approaches by Kong as well as several other researchers [1], [2], [24], [25] by reconstructing between-the-measurements HPV statuses (i.e., presence or absence of HPV infection). This approach allows for inclusion of the whole longitudinal dataset, thus increasing the accuracy of prediction of probability of HPV clearance without making multiple assumptions about how the time of incidence, clearance, and censoring events could be reconstructed (as it is required for Kaplan-Meier and Cox analyses). The shorter intervals accessible in our method allow for taking into account the dynamics of potential predictors, which could change quickly (such as CD4 count, HIV-1 VL, and HAART regimen). This model allows for calculating the clearance probability with subsequent confirmation in another 3 months—
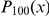
—by transferring the probabilities such as 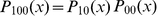, thus corresponding to the routine definition of HPV clearance when the absence of HPV type-specific infection is required for two subsequent visits. Opposite to the Cox model, in which HRs are estimated for time-dependent covariates, the developed approach allows us to estimate the transition probability and evaluate its standard errors. Since the developed model provides the hazard function for probability of HPV infection clearance, the respective survival function and characteristics of time-to-clearance distribution also can be evaluated: e.g., time to clearance (in months) could be estimated as 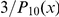, and a median of HPV clearance time as 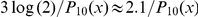. The approaches utilizing the generalized estimating equation (GEE)—they take into account the mutual correlations in clearance of different HPV types and modeling mixed effects, allowing individuals to have their own characteristics (i.e., distributed in a population)—could be used to further enrich the developed base model; Xue et al. [26] recently reviewed the series of approaches that can be used for similar generalizations.

In both approaches, a transitional probability-based model and Cox regression model, CD4 count was a significant predictor of clearance of all phylogenetic HPV groups in HIV-1-infected adolescent females; also, certain effects of HAART (with PI) on clearance of HPV16/16-like and HPV18/18-like infections were observed. However, while in the Cox model, being HIV-1-infected had a minor effect only on HPV56/56-like clearance, in the transitional probability model, this factor was a significant predictor of clearance of HPV16/16-like, HPV56/56-like, and low-risk HPVs.

In immunodeficient patients, the mechanisms by which immune deficiency increases the risk of persistence of HPV infection are still poorly understood: some alterations in dendritic antigen-presenting cells, Langerhans cells, and macrophages function, as well as a deficient cytotoxic lymphocyte response to E6 and E7 proteins, might be the contributing factors [27], [28]. The results obtained from our study about the role of CD4 in HPV clearance corroborate previous reports from the REACH cohort, as well as several other studies on adult HIV-infected females [1], [2], [29], [30], [31], [32], [33], [34]. However, there is no agreement about the role CD4 play in clearance of individual types of HPVs; e.g., in several studies on both HIV-1-negative and HIV-1-positive females, it has been shown that HPV16 infection has a lower probability of clearance than other HPV types, possibly due to its greater ability to escape immunologic surveillance [35], [36], [37], while other studies did not demonstrate such a difference [38]. In our study, a lower clearance probability was registered for the HPV16/16-like than for the 18/18-like group, while HPV16 had an equal-with-HPV18 chance to be cleared at both pathologic and normal CD4 counts. The observed heterogeneity of phylogenetic groups of HPV infection in terms of a probability of HPV clearance may depend not only on CD4 counts and other predictors measured at current time (such as HIV-1 VL and HAART with PI), but also from the history of HPV type-specific infection (e.g., from the time since HPV acquisition, which is an unobserved variable), depending on a prevalent or incident type-specific HPV infection. Further analysis could be performed using non-Markov approaches to model unobserved time since HPV acquisition.

HIV infection, independent of CD4 count, has also been suggested to be a predictor of persistence of HPV infection in HIV-1-positive women. This may imply an alternate mechanism besides CD4, e.g., via alteration of the cytokine response to HPV infection in the cervical mucus [2], [39], [40], [41], [42]. In our study, being HIV-1-positive affected the probability of clearance of HPV16/16-like, 18/18-like, and low-risk HPVs. In the REACH cohort–based study by Moscicki et al. [2], when only subjects with normal CD4 counts (i.e., ≥500 cells/mm^3^) were considered, the multivariable regression showed high significance of HIV status as an independent predictor of HPV clearance event (HR = 1.60, p = 0.012).

Currently, prognostic importance of high HIV-1 VL for HPV clearance is not clear, but it likely increases the risk of persistence of HPV infection at low CD4 cell counts [32], [43]. In our study, HIV-1 VL could affect the clearance of low-risk HPVs and certain oncogenic HPVs (e.g., HPV58 and HPV59). The apparent impact of HAART on HPV incidence, clearance, and persistence also is not clear [11], [23], [32], [44], [45]. In our study, when HAART was analyzed taking into account its PI component, a significant effect was observed for HPV16, and minor effects were observed for HPV16/16-like, HPV18/18-like, and HPV59. In vitro studies have shown that specific PIs inhibit the ability of HPV16 E6 to degrade p53 and selectively kill E-6-dependent cervical carcinoma cells [46]. Previous crossover analyses in REACH suggested no significant effect of HAART on HPV clearance [11]; however, the effect of PI was not examined, as it is incorporated in this new method. These results require further investigation with longer follow up and more detailed analysis of HAART/PI history and dose/exposure.

The observation on *C. trachomatis* increasing probability of clearance of low-risk HPV falls in with the results from animal studies about potential role of interferon-γ as local “protector” against other (i.e., non-*Chlamydia*) infections [47], [48]. Oncogenic HPV types could be strong enough to avoid this mechanism; recently, it has been speculated that *C. trachomatis* could have effect on oncogenic HPV types [49].

The results of this study have several limitations. While the prevalence and incidence of HPV infections among HIV-1-positive adolescents in the REACH study is high [11], some of the associations may have been limited by the relatively smaller sample size of HIV-1-negative individuals along with the lower HPV infection rate. Due to the populations served at the REACH recruitment sites, young African-American women were a significant proportion of the population; therefore, the results may not be fully generalized to other populations. Also, the interrelations described in this study were obtained on a cohort of young adolescent girls with relatively short histories of HIV-1 infections, who are generally healthy and whose immune response to the infection may differ from older women; for example, it has been shown in several studies that older age was associated with higher risk of HPV persistence in both HIV-infected and HIV-uninfected women [24], [50]. Regarding the approach, the simple version of the model was intentionally chosen as a base model, resulting in some limitations; e.g., there was no distinction between the effects of incident infection and re-infection, no correlations between clearance of distinct HPV types in one individual were modeled, and the time after the incidence was not explicitly represented. Due to the two-step design of the study, some variables which were statistically insignificant were not included into the second step of the analysis thus potentially compromising the robustness of the model. Nevertheless, the limitations can be overcome by the extensions of the proposed approach using approaches specifically developed for analyses of HPV clearance [26] and those that were successfully used in other related research areas, e.g., g-formula [16] or a (binomial) stochastic process model [51], [52], [53].

In summary, our new model estimates a probability for HPV clearance of type-specific HPV groups at a 3-month period by coordinating uneven time scales of measurements on biannual HPV status and other quarterly HIV-1-related clinical data and risk factors.

## Supporting Information

Table S1 HPV type-specific characteristics obtained from the M1 – M7 models tested in the REACH cohort of HIV-1-positive and HIV-1-negative adolescent girls enrolled in 1996-2000.
(DOC)
