# Supplementary material for: Transitional Probability-Based Model for HPV Clearance in HIV-1-Positive Adolescent Females
Source: PLoS One. 2012 Jan 24;7(1):e30736. doi: 10.1371/journal.pone.0030736 (PMC3265500; doi:10.1371/journal.pone.0030736)
Supplement: Table S1 — HPV type-specific characteristics obtained from the M1 – M7 models tested in the REACH cohort of HIV-1-positive and HIV-1-negative adolescent girls enrolled in 1996-2000. (DOC) [file pone.0030736.s001.doc]

Supplemental Table. HPV type-specific characteristics obtained from the M1 – M7 models tested in the REACH cohort of HIV-1-positive and HIV-1-negative adolescent girls enrolled in 1996-2000.

| **HPV type** | **Model** | **HIV**  **status** | **Main parameters in the model** | | | |  | **Additional parameter in the model** | | |
| --- | --- | --- | --- | --- | --- | --- | --- | --- | --- | --- |
|  |  |  | *u_00_* (±SE) | *β_00_* (±SE)^a^ | *u_11_* (±SE) | *β_11_* (±SE)^a^ |  | Type of parameter | Estimate (±SE) | log likelihood |
| **16/16-like** | M1 | HIV(-) | -3.7±0.47** | -0.24±0.52 | -0.13±0.58 | -0.27±0.64 |  | Basic model | –– | -400.50 |
|  |  | HIV(+) | -3.5±0.15** | -0.14±0.25 | -1.52±0.15** | 1.15±0.27** |  |  | –– | -1405.67 |
|  | M2 | both | -3.44±0.13** | -0.36±0.2* | -1.1±0.25** | 0.9±0.23** |  | HIV-1/intercept effect | -0.31±0.19* | -1809.87 |
|  | M3 | both | -3.44±0.13** | -0.36±0.2* | -1.4±0.14** | 1.16±0.23** |  | HIV-1/CD4 effect | -0.2±0.22 | -1810.83 |
|  | M4 | HIV(+) | -3.49±0.14** | -0.14±0.25 | -1.55±0.02** | 1.26±0.33** |  | CD4 squared^b^ | -0.09±0.37 | -1405.65 |
|  | M5 | HIV(+) | -3.49±0.15** | -0.14±0.25 | -1.84±0.36** | –– |  | Piecewise interpolation | *β*_200_=-1.12±0.22**  *β*_500_=-0.98±0.17**  *β*_800_=-0.62±0.2**  *β*_1500_=0.24±0.51 | -1405.07 |
|  | M6 | HIV(+) | -3.47±0.17** | -0.09±0.28 | -0.7±0.47* | 0.79±0.35** |  | HIV VL effect | -0.17±0.09* | -1091.49 |
|  | M7 | HIV(+) | -3.5±0.14** | -0.14±0.25 | -1.65±0.18** | 1.23±0.27** |  | HAART/PI effect | 0.33±0.18* | -1404.64 |
| **18/18-like** | M1 | HIV(-) | -3.96±0.48** | -0.12±0.53 | -0.15±0.66 | 0.24±0.71 |  | Basic model | –– | -377.42 |
|  |  | HIV(+) | -3.38±0.14** | -0.47±0.25* | -1.29±0.18** | 1.58±0.36** |  |  | –– | -1428.85 |
|  | M2 | both | -3.36±0.12** | -0.61±0.2** | -0.95±0.32** | 1.25±0.31** |  | HIV-1/intercept effect | -0.21±0.24 | -1809.93 |
|  | M3 | both | -3.36±0.12** | -0.62±0.2** | -1.19±0.7** | 1.41±0.29** |  | HIV-1/CD4 effect | -0.05±0.28 | -1810.31 |
|  | M4 | HIV(+) | -3.37±0.13** | -0.48±0.24** | -1.4±0.23** | 2.12±0.81** |  | CD4 squared^b^ | -0.5±0.64 | -1428.59 |
|  | M5 | HIV(+) | -3.38±0.13** | -0.48±0.25* | -1.89±0.39** | –– |  | Piecewise interpolation | *β*_200_=-0.55±0.21**  *β*_500_=-0.78±0.18**  *β*_800_=0.29±0.27  *β*_1500_=0.54±0.63 | -1425.45 |
|  | M6 | HIV(+) | -3.48±0.16** | -0.2±0.28 | -0.98±0.64* | 2.1±0.66** |  | HIV VL effect | -0.14±0.12 | -1074.58 |
|  | M7 | HIV(+) | -3.38±0.13** | -0.47±0.25* | -1.38±0.18** | 1.58±0.36** |  | HAART/PI effect | 0.29±0.21* | -1428.51 |
| **56/56-like** | M1 | HIV(-) | -2.8±0.68** | -0.74±0.77 | 0.51±1.03 | -0.21±1.12 |  | Basic model | –– | -186.22 |
|  |  | HIV(+) | -2.82±0.19** | -0.59±0.35* | -0.99±0.22** | 0.72±0.38* |  |  | –– | -695.73 |
|  | M2 | both | -2.79±0.18** | -0.68±0.28** | -0.14±0.4 | 0.61±0.36* |  | HIV-1/intercept effect | -0.81±0.32** | -882.38 |
|  | M3 | both | -2.8±0.18** | -0.66±0.29** | -0.93±0.22** | 1.47±0.47** |  | HIV-1/CD4 effect | -0.87±0.4** | -883.24 |
|  | M4 | HIV(+) | -2.82±0.19** | -0.58±0.34* | -1.21±0.31** | 1.65±1.05 |  | CD4 squared^b^ | -0.74±0.78 | -695.25 |
|  | M5 | HIV(+) | -2.81±0.19** | -0.59±0.35* | -1.46±0.48** | –– |  | Piecewise interpolation | *β*_200_=-0.65±0.32**  *β*_500_=-0.7±0.24**  *β*_800_=-0.24±0.33  *β*_1500_=-0.38±0.66 | -694.85 |
|  | M6 | HIV(+) | -3.05±0.24** | -0.35±0.4 | -0.9±0.67* | 0.7±0.51* |  | HIV VL effect | -0.05±0.13 | -498.26 |
|  | M7 | HIV(+) | -2.82±0.19** | -0.58±0.35* | -1.03±0.25** | 0.74±0.38** |  | HAART/PI effect | 0.09±0.25 | -695.71 |
| **Low risk** | M1 | HIV(-) | -5.3±0.59** | 0.93±0.61* | -1.28±1.22* | 2.08±1.46* |  | Basic model | –– | -251.76 |
|  |  | HIV(+) | -3.69±0.15** | -0.26±0.27 | -1.31±0.21** | 1.5±0.41** |  |  | –– | -1210.89 |
|  | M2 | both | -3.67±0.14** | -0.48±0.22** | -0.33±0.43 | 1.32±0.37** |  | HIV-1/intercept effect | -0.94±0.35** | -1469.03 |
|  | M3 | both | -3.68±0.14** | -0.47±0.23** | -1.26±0.2** | 2.38±0.47** |  | HIV-1/CD4 effect | -1.06±0.43** | -1469.56 |
|  | M4 | HIV(+) | -3.66±0.15** | -0.31±0.26 | -1.66±0.29** | 3.1±1.01** |  | CD4 squared^b^ | -1.34±0.74* | -1209.37 |
|  | M5 | HIV(+) | -3.65±0.15** | -0.33±0.26 | -1.53±0.5** | –– |  | Piecewise interpolation | *β*_200_=-1.37±0.27**  *β*_500_=-0.01±0.24  *β*_800_=-0.45±0.31  *β*_1500_=0.41±0.7 | -1206.94 |
|  | M6 | HIV(+) | -3.82±0.17** | 0.05±0.29 | -0.13±0.64 | 0.93±0.51* |  | HIV VL effect | -0.26±0.13** | -926.94 |
|  | M7 | HIV(+) | -3.69±0.15** | -0.27±0.27 | -1.42±0.22** | 1.54±0.41** |  | HAART/PI effect | 0.33±0.24 | -1210.86 |

Notes: * - 0.05≤p<0.1; ** - p<0.05.

^a^ – the units of *β_00_* and *β_11_* are 1000/[C], where [C] is the unit of CD4 cell counts, i.e., cell/mm^3^; ^b^ - the unit of additional parameter in the M4 model is 1000000/[C]^2^, where [C] is the unit of CD4 cell counts, i.e., cell/mm^3^.
